# Numerical Study of the Mechanical Behaviour of Wedge-Shaped Defect Filling Materials

**DOI:** 10.3390/ma15207387

**Published:** 2022-10-21

**Authors:** Lyaysan Sakhabutdinova, Anna A. Kamenskikh, Alex G. Kuchumov, Yuriy Nosov, Inessa Baradina

**Affiliations:** 1 Department of Computational Mathematics, Mechanics and Biomechanics, Perm National Research Polytechnic University, 614990 Perm, Russia; 2 Clinics “Profi–Dent”, 220013 Minsk, Belarus

**Keywords:** tooth, NCCL, contact, modelling, FEM, biomaterials, strain

## Abstract

This paper deals with direct restorations of teeth with non-carious cervical lesions (NCCL). NCCL defects are capable of gradual growth and are accompanied by the degradation of the surrounding tissue. Direct restorative treatment, in which the cavity is filled with a cementing agent, is considered to be an accessible and common treatment option. The study included simulations of the teeth without lesions, the teeth with V and U lesions and the tooth-restorative system. Parameterised numerical tooth models were constructed. Two cases with defect depths of 0.8 mm and ~1.7 mm and three variants with fillet radii of the defect end of 0.1, 0.2 and 0.3 mm were considered. The effect of two biomaterials for restorations was studied, namely Herculite XRV (Kerr Corp, Orange, CA, USA) and Charisma (Heraeus Kulzer GmbH, Hanau, Germany). The models were deformed with a vertical load of 100 to 1000 N from the antagonist tooth. The tooth-restorative system was considered, taking into consideration the contact interaction in the interface areas with the tooth tissues. Within the limits of the research, the character of the distribution of the deformation characteristics and their dependence on the level of loading, the depth of the defect and the radius of the curvature of the “wedge” were established.

## 1. Introduction

### 1.1. Problem Context

A wedge-shaped defect occurs after damage to the dental tissue near the neck of the tooth, and it is shaped like a wedge, with its base being in the enamel and its apex being in the dentin. A wedge-shaped defect is a case of a non-carious cervical lesion (NCCL). NCCLs have been reported [1,2,3] to occur in up to 40–60% of the adult population, depending on their age and region of residence. Despite the non-carious nature of these lesions, clinical defects are progressive if they are left untreated [4,5]. Previously, erosion and abrasion were the main causes of wedge defects. Today, scientists are increasingly investigating the biomechanical causes of NCCLs. Occlusal loading has been identified as the main mechanical factor in the occurrence and development of wedge-shaped NCCLs [6,7].

Dentists distinguish between two types of NCCLs according to the shape of the “wedge” section: V and U differential. Studies show that V-shaped wedge defects increase the stress concentration by a factor of four in comparison to that of the U-shaped ones at the floor of the cavity under the same loading conditions [8]. In addition to the shape of the wedge-shaped defect, the lesion depth has a significant influence on the stress–strain state. It was demonstrated in [9] that as the lesion spreads deeper into the tooth, the stresses increase in the area of the apex of the “wedge”.

Several restoration options are available to prevent the enlargement of the wedge defect. Doctors offer both of the standard fillings which are those that are close the cavity of the defect and the newer approaches that are in the form of special ceramic inlays [10,11]. Nowadays, the restoration of the wedge-shaped defect cavity with cementitious compositions remains a common method [12]. However, such restorations are often not durable and need to be replaced frequently [13,14,15]. Dental adhesive glues are often used to extend the life of restorations [16,17]. Several studies have investigated the effect of cavity preparation prior to the insertion of the restoration [18]. It is also worth mentioning that etching compounds have been used to increase the adhesion between the restorations and the dental tissues. Measures to increase the adhesion between the restorations and the tooth substance do not, however, address the individual mechanical reasons for the short lifespan of the fillings in the treatment of NCCLs. Today, choosing the optimal filling material for a specific clinical case is still an important issue.

### 1.2. Problem Description

The mechanical causes of the occurrence and development of wedge-shaped defects can be investigated from the perspective of deformable solid mechanics. Finite element analysis (FEM) tools are well suited for this purpose. FEM has become firmly established in the medical practice of solving dental problems [19,20,21,22,23]. FEM also makes it possible to evaluate the effectiveness of different treatment tactics and material choices at the treatment planning stage [24].

Different materials are selected depending on the depth of the NCCL [25]. Wedge-shaped defects that are up to 0.5 mm in depth with a high risk of rapid deepening are filled with liquid–fluid composites. Liquid–fluid materials are used because of their high elasticity, which allows for the partial compensation of the pathological occlusal load of the antagonist teeth [26,27]. Two groups of materials are used for the treatment of defects that are greater than 0.5 mm in depth: glass ionomer cement and microfill composites [28,29,30].

Studies have shown that restorations that are made of glass ionomer cements have a fine network of cracks in the area that is adjacent to the dental hard tissue, which are then filled with stains [31]. These cracks form when the antagonist tooth remains under traumatic stress. The filling, therefore, becomes unaesthetic by the end of the first year if it is in the smile zone. Microfill composite materials have good aesthetic and wear resistance properties [32]. On the other hand, their polymerisation shrinkage rates are high. In addition, cracks may develop in the interface between the filling material and the tooth substance if the traumatic occlusion load of the antagonist is maintained [33,34].

A filling technique that reduces the negative qualities of each of the materials that has been described has also become widespread. The idea is that the lower or inner part of the filling is made of glass ionomer cement and the upper part of the filling is made of composite material [35,36].

In the future, studies on the mechanical behaviour of restorations that are made from different materials will provide evidence-based recommendations for practicing dentists on their choice of treatment tactics. At this stage in this study, it was decided to investigate the deformation behaviour of the tooth with restorations using two common cement alternatives.

The study involved modelling the tooth deformation problem with and without the wedge-shaped defects, as well as modelling the direct restoration of a wedge-shaped defect. The simulations were performed in the ANSYS Mechanical APDL application package (ANSYS Inc., Canonsburg, PA, USA). The tooth geometry was modelled in the first approximation, and it was more rounded. The crown geometry was able to be changed. In the first approximation, the crown geometry was not symmetrical, and it had different heights, i.e., an attempt was made to approximate the crown geometry to the individual case.

### 1.3. Research Objectives

The object of the study was to perform a direct restoration of a wedge-shaped defect that closes a cavity. The aim of the study was to analyse the effect of the restorative materials on the biomechanical deformation of the tooth-denture system.

Research objectives:

- The creation of parameterised tooth models with and without a consideration of the wedge-shaped defect, including models with the defect deepening of the pulp;
- The implementation of a series of numerical experiments on the deformation of the tooth-fillings system at different loading levels;
- An analysis of the influence of the restorative materials on the deformation of the biomechanical system in a wide range of loading conditions from the antagonist tooth.

## 2. Materials and Methods

### 2.1. Design of the Experiment

**Figure 1:**
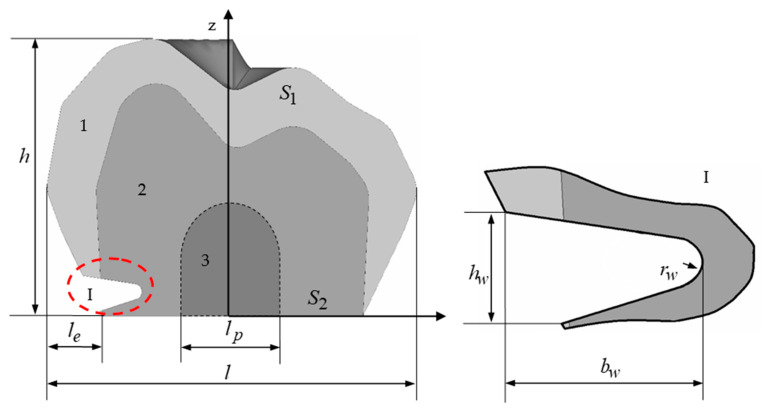
Mid-section of a tooth with a wedge-shaped defect.

As part of the study, a parameterised tooth model of the wedge-shaped defect was created. The nonlinearity of the geometric configuration of the wedge-shaped defect was taken into account. The central cross-section of the tooth with the defect is shown in Figure 1. The tooth model included the volume of the enamel (1) and dentin (2). The pulp (3) was included in the geometry of the wedge defect, but it was not modelled in the calculation scheme.

The tooth model did not include the roots; the discarded part of the tooth was replaced by the boundary conditions. Parameterisation of the tooth geometry was performed on its main parameters: height h, length l and enamel thickness $l_{e}$ (the enamel thickness was specified as a parameter; the actual enamel thickness of the tooth in the model varied within $l_{e}$, i.e., it may have varied more or less by 10–15% $l_{e}$). Figure 1 shows the geometric configuration of the tooth with a height h≈7.2 mm, length l≈9.44 mm and enamel thickness $l_{e}\approx 1.5$ mm. Overall, the dimensions of the tooth corresponded to the premolars and molars and fell within the range of acceptable values [6]. All of the dimensions were parameterised, and they were able to be changed. Thus, calculations were able to be made for different values of the tooth parameters. Static boundary conditions were given (the force from the antagonist tooth) on the tooth surface $S_{1}$; kinematic conditions were given (the force from tooth that was fixed against the normal) on the tooth surface $S_{2}$.

A wedge-shaped defect was created using a triangle, part of which intersected with the tooth on the left side. The auxiliary triangle was parameterised using the position and coordinates of the three nodes. The corner of the triangle, which was located in the dentin of the tooth at a radius of curvature $r_{w}$, was rounded. Figure 1 shows the model with a radius of $r_{w}=0.2$ mm. The model was parameterised for the fillet of the wedge-shaped defect, i.e., the model was able to be changed automatically when the fillet radius was changed.

To cut out the volume of a wedge-shaped defect, its mid-section was extended along the *z*-axis. The simulation resulted in a wedge-shaped defect of $2l_{w}\times h_{w}\times b_{w}$. Two different wedge depths $b_{w}$ were investigated: the first variant had a wedge depth of 0.9 mm, and the second variant had a wedge depth of approximately 0.8 mm in dentin. A range of fillet radii were considered for the wedge defects that were between 0.1 and 0.3 mm in 0.1 mm increments.

### 2.2. Mechanical Properties of the Materials

The materials of the model were considered in an elastic formulation. The modulus of elastic compression (*E*) and Poisson’s ratio (v) of the enamel and dentine are given in Table 1. The properties of the dentin and enamel were taken from the reference literature [8].

Two options were considered for standard restorations: Herculite XRV microfine composite (Kerr Corp, Orange, CA, USA) was material 1, and Charisma glass ionomer cement (Heraeus Kulzer GmbH, Hanau, Germany) was material 2. The physical and mechanical properties of the inlay materials are presented in Table 2. The properties of the restorative materials were taken from the reference literature [35,37]. The materials that were under consideration are widely used in dental practice.

### 2.3. Loading and Boundary Conditions

The mathematical problem statement is described in [38], and it includes equilibrium equations and physical and geometric relations, as well as contact boundary conditions. The task was implemented as a 3D problem. The mathematical formulation of the problem was supplemented by boundary conditions: a constant load varying from 100 to 1000 N (indentation force) was applied at the boundary S_1_; at the boundary S_2_, movement along the vertical coordinate *y* was prohibited. The boundary of S_2_ region was additionally constrained by horizontal displacement and angular rotation to imitate a parodont connection.

### 2.4. Numerical Finite Element (FE) Solution and Convergence

The simulation was implemented using the applied ANSYS Mechanical APDL engineering analysis package (ANSYS Inc., Canonsburg, PA, USA) by the finite element method using volume finite elements SOLID185 (four-node tetrahedra) with a Lagrangian approximation and three degrees of freedom at each node. Contact gluing was modelled in the inlay–tooth interface zones, taking into account friction.

The model eliminates account the divergence of account the contact surfaces, as well as appearance of slip zones. Contact interaction was modelled using a contact pair of elements (CONTA173 and TARGE170). The surface–surface contact was considered. The contact algorithm was the extended Lagrange method.

**Figure 2:**
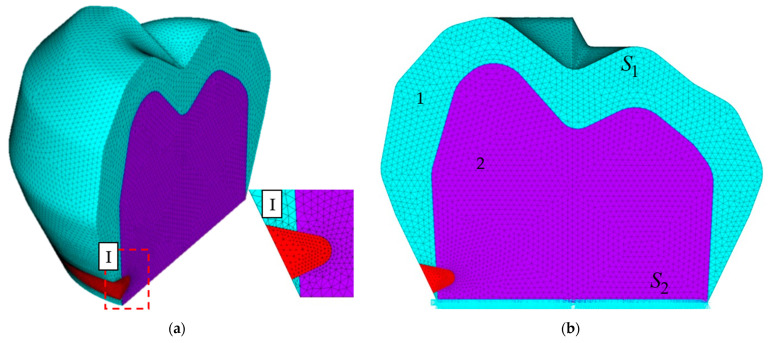
FE model: (**a**) 3D cross-section, and the (**b**) section with boundary conditions.

Figure 2 contains the finite element model. As a result of a series of computational experiments, the convergence of the numerical procedures for the strain and contact characteristics was established. A finite element partitioning with ~545 k nodal unknowns was chosen. A mesh densification near the wedge-shaped defect and the seal was performed. The maximum element dimension was 0.15 mm, and the minimum one was 0.05 mm. The finite element mesh was chosen within the assessment of the influence of the system discretisation degree on the numerical solution of the problem. The minimum overall dimension of the finite element near the tooth–inlay contact area was 0.05 mm. When moving away from the contact zone, the size of the finite elements increased in a gradient. The maximum overall dimension of the final element reached 0.15 mm.

## 3. Results

### 3.1. Stress–Strain Analysis of the Biomechanical Unit for a Shallow Wedge-Shaped Defect

**Figure 3:**
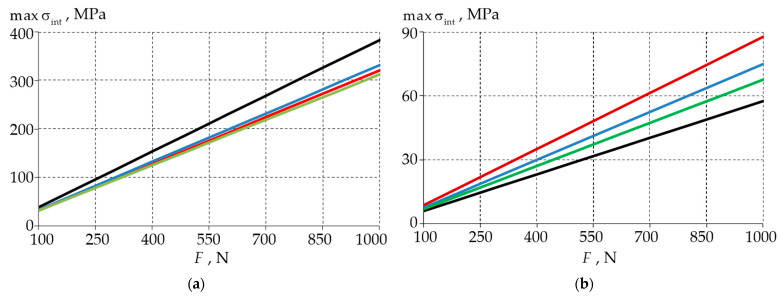
Dependence of maximum stress intensity values of the biomechanical unit on load: (**a**) enamel; (**b**) dentin; black—model without defect; red—model with 0.1 mm defect fillet; blue—model with 0.2 mm defect fillet; green—model with 0.3 mm defect fillet.

The first step was to analyse the stress–strain state of the biomechanical unit with a shallow wedge-shaped defect. The maximum stress intensity in the enamel and dentin was preliminarily investigated as a function of the level of the external loading for a healthy tooth and three fillet options (Figure 3).

The presence of a wedge-shaped defect reduced the enamel stress intensity by 13–18%. The value of the radius of the fillet had an insignificant effect on the maximum stress values. The lowest stress values were observed in the wedge-shaped defect with a fillet of 0.3 mm. The stress intensity was higher in the teeth without defects. This was because the enamel absorbed most of the force of the external load in the area of the kinematic boundary conditions. In the presence of a defect, the maximum stresses were displaced to the upper boundary of the wedge.

The opposite was true of the dentin. The presence of the defect led to an increase in the stress intensity. At a radius of 0.1 mm, a 1.5-fold increase in the maximum values of $\sigma_{\mathrm{int}}$ was observed. At the fillet radius of 0.3 mm, the increase was 17%. This is explained by the presence of a stress concentrator in the dentin at the end of the wedge defect. The sharper the wedge was, then the higher the stress was.

**Figure 4:**
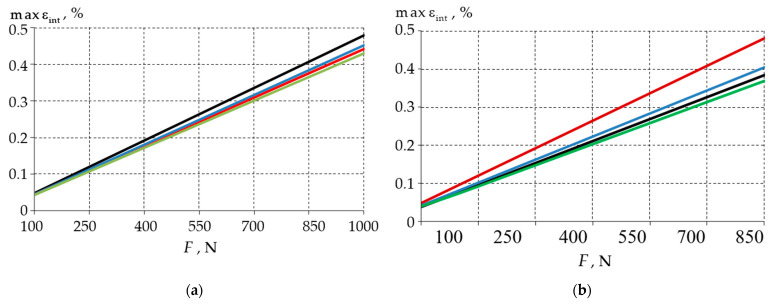
Dependence of maximum strain intensity values of the biomechanical unit on load: (**a**) enamel; (**b**) dentin; black—model without defect; red—model with 0.1 mm defect fillet; blue—model with 0.2 mm defect fillet; green—model with 0.3 mm defect fillet.

Figure 4 shows the dependence of the maximum strain rate on the load level.

For the maximum deformation rates in the enamel, a reduction by 5–10% was observed in the presence of the defect. The largest decrease, by 10%, was in the defect with a 0.3 mm curvature radius. The maximum increase in the strain rate values in the dentin was 25% in the defect with a fillet radius of 0.1 mm. However, in the 0.3 mm radius rounded defect, a 3% decrease in the maximum values of $\varepsilon_{\mathrm{int}}$ was observed when it was compared to the whole tooth. This result is explained by the shifting of the local maxima from the boundary conditions zone to the zone of the wedge-shaped defect fillet.

**Figure 5:**
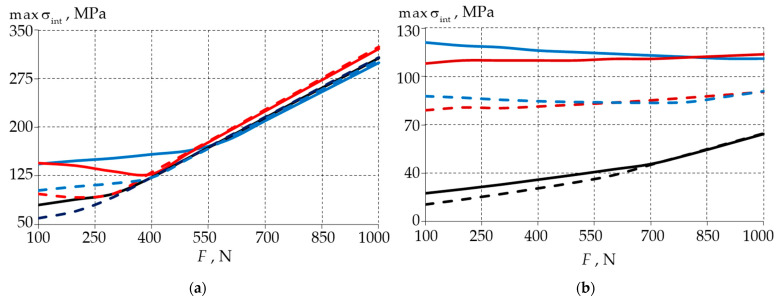
Maximum stress intensity of a biomechanical unit as a function of load: (**a**) enamel; (**b**) dentine; dashed line—model with filling material 1; solid line—model with filling material 2; blue—model with 0.1 mm fillet; red—model with 0.2 mm fillet; black—model with 0.3 mm fillet.

In the second step, two models of the biomechanical unit with standard restorations in two materials were considered. Figure 5 shows the maximum $\sigma_{\mathrm{int}}$ in the enamel and dentin dependencies for the two restorative materials.

The nature of the maximum stresses was divided into two zones. The first zone involved a gradual increase in the parameters, and in this zone, the maximum values were observed in the wedge-shaped defect. The second zone was a zone involving a steep increase in the parameters, and in this zone, the maximum of the parameters was shifted to the cervical area. The maximum intensities of the stresses at a force of more than 500 N from the antagonist tooth had small differences and increased in proportion to the load.

In the analysis of the relationship, it was observed that the maximum stress values of material 1 (Herculite XRV, Kerr Corp, Orange, CA, USA) were lower. In enamel, the stresses were independent of the choice of restorative material from a load of 550 N. The greatest difference was observed in the load range of 100 to 250 N. The maximum stress was observed in the case of the defect with a 0.1 mm fillet.

In the dentin, using material 1, the stress rate was 30% lower for the 0.1 mm and 0.2 mm rounded defects. In the case of the defect with a fillet radius of 0.3 mm, the choice of material had no significant effect on the stress level.

**Figure 6:**
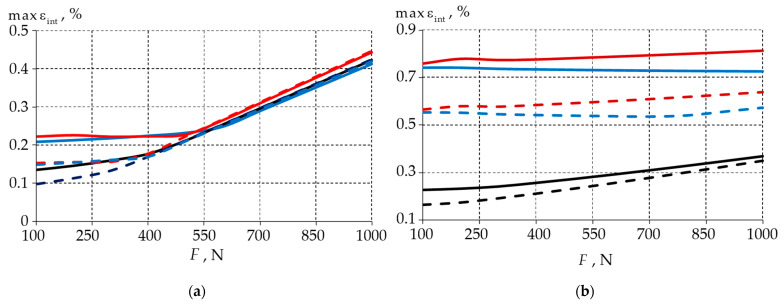
Maximum strain intensity of a biomechanical unit as a function of load: (**a**) enamel; (**b**) dentine; dashed line—model with filling material 1; solid line—model with filling material 2; blue—model with 0.1 mm fillet; red—model with 0.2 mm fillet; black—model with 0.3 mm fillet.

Figure 6 shows the dependencies of the maximum strain intensity on the load level.

The maximum strain rate dependencies repeated the trends that were found in the stress analysis. For a load of 100 N, the deformation in the enamel with a 0.3 mm defect was 50% lower than it was in the models with the 0.1 and 0.2 mm fillets. In the dentin, the deformations for a 0.3 mm fillet were 70% lower than they were for a 0.1 mm fillet. The use of material 1 for the wedge-shaped defects reduced the maximum $\varepsilon_{int}$ in the dentin by an average of 30%.

**Figure 7:**
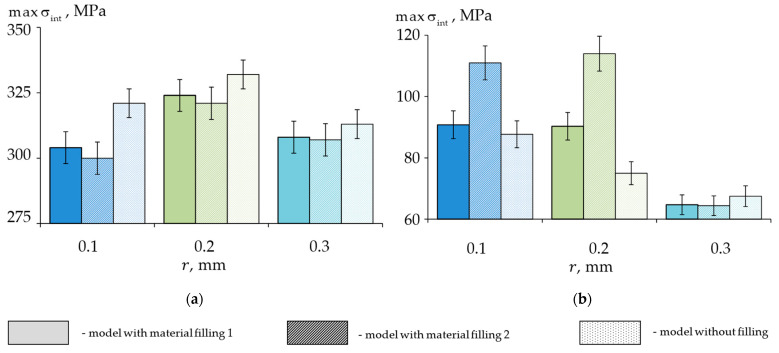
Dependence of the maximum stress intensity of the biomechanical unit on the fillet radius of a shallow wedge-shaped defect: (**a**) enamel; (**b**) dentin.

**Figure 8:**
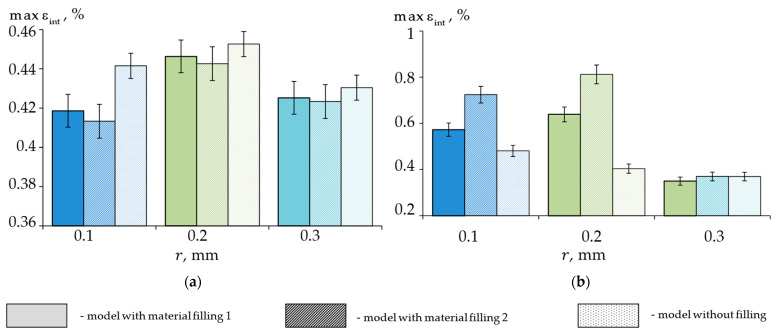
Dependence of the maximum strain intensity of the biomechanical unit on the fillet radius of a shallow wedge-shaped defect: (**a**) enamel; (**b**) dentin.

To assess the effect of the fillings on the stress–strain state of the biomechanical unit, a comparison of the maximum stress and strain intensities under a 1000 N load in the enamel and dentin for the three calculated models is presented in Figure 7 and Figure 8, respectively.

A reduction in maximum stresses was obtained in the wedge defect with a standard filling when it was compared to those of the unrestored model. A minimum σ_int_ value was obtained in the enamel with material 2. The most significant reduction in the stress levels was observed with a defect radius of 0.1 mm. The difference between the stress levels of the different restorative materials was in the range of 2%.

In the dentin, an increase in σ_int_ was observed for the 0.1 and 0.2 mm fillets in the defect restorations. The stress level when we were using material 1 as a restoration was 20% lower when it was compared to that of material 2. In the model with a 0.3 mm defect fillet, there was a slight decrease in σ_int_ when it was compared to that of the case without a restoration.

The distributions of the maximum strain intensities at 1000 N were similar to those of the stresses. As a result of the simulation, it was found that the use of standard fillings for the wedge-shaped defects with a fillet radius of 0.3 mm reduced the maximum stresses and strains in the biomechanical unit, and the choice of material was not critical. With the 0.1 and 0.2 mm radii, standard fillings were found to reduce the stresses and strains in the enamel, but these increased by 20–50% in the dentine. The use of Herculite XRV (Kerr Corp, Orange, CA, USA) was preferable in terms of the overall stress–strain analysis of the biomechanical unit.

### 3.2. Comparison of the Stress–Strain State of the Biomechanical Unit for a Shallow and Deep Wedge-Shaped Defect

**Figure 9:**
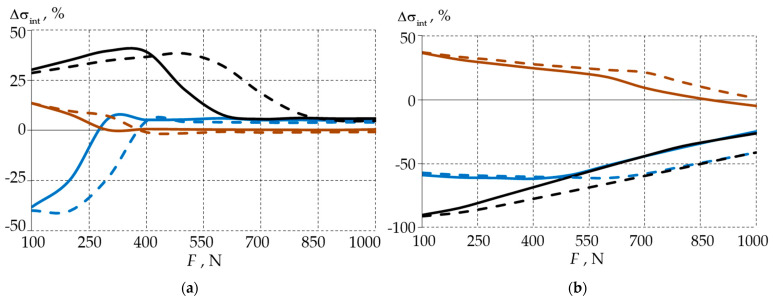
Comparison of biomechanical stress intensities of a shallow and deep wedge defect: (**a**) enamel; (**b**) dentin; solid line—model with filling material 1; dotted line—model with filling material 2; black—model with 0.1 mm rounded defect, blue—model with 0.2 mm rounded defect, brown—model with 0.3 mm rounded defect.

**Figure 10:**
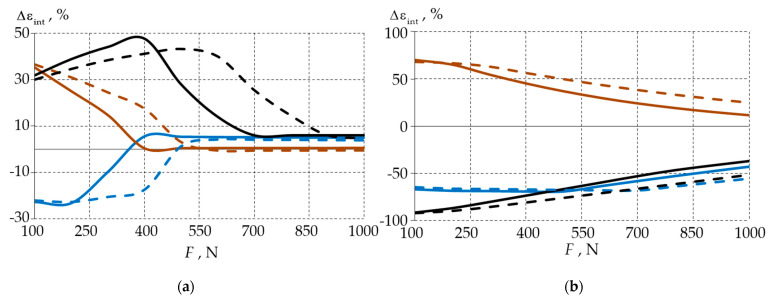
Comparison of the deformation intensities of the biomechanical unit of a deep versus shallow wedge defect: (**a**) enamel; (**b**) dentin; solid line—model with filling material 1, dashed line—model with filling material 2; black—model with 0.1 mm fillet, blue—model with 0.2 mm fillet, brown—model with 0.3 mm fillet.

In order to investigate the influence of the filling material on the deformation behaviour of the biomechanical unit in more detail, the deviations in the maximum stress and strain rates of the model with a deep wedge-shaped defect were compared to those of the model with a shallow defect. Figure 9 and Figure 10 show the deviations in the maximum stress $\Delta \sigma_{\mathrm{int}}$ and the maximum strain $\Delta \varepsilon_{\mathrm{int}}$ from the level of the external load.

To compare the influence of the wedge depth on the deformation characteristics of the tooth-filling system, we analysed the stress intensity using Formula (1) and the deformation intensity using Formula (2), where the values of the deformation parameters of the shallow wedge model were taken as reference values:

(1) $$\Delta \sigma_{\mathrm{int}}=\left({\left.\max\sigma_{\mathrm{int}}\right|}_{b_{w}\approx 0.9}-{\left.\max\sigma_{\mathrm{int}}\right|}_{b_{w}\approx 1.7}\right)/{\left.\max\sigma_{\mathrm{int}}\right|}_{b_{w}\approx 0.9}\cdot 100\%,$$

(2) $$\Delta \varepsilon_{\mathrm{int}}=\left({\left.\max\varepsilon_{\mathrm{int}}\right|}_{b_{w}\approx 0.9}-{\left.\max\varepsilon_{\mathrm{int}}\right|}_{b_{w}\approx 1.7}\right)/{\left.\max\varepsilon_{\mathrm{int}}\right|}_{b_{w}\approx 0.9}\cdot 100\%.$$

In tooth enamel, a significant increase in the stress intensity was realised in the defect with a fillet radius of 0.1 mm. For the deep wedge and the fillet radius of 0.2 mm, the stresses were lower than those with the shallow defect. At a 0.3 mm radius, the increase was 15% under the 100 N load; further, the depth of the wedge had little effect on the σ*_int_* values. In material 1, the deviations were smaller for the 0.2 and 0.3 mm fillets, and under the loading level of 400 N, the stress levels in enamel were minimal, indicating that for this material, the stress level in the deep wedge was lower.

In the dentin, in the case of the deep wedge with a curvature radius of 0.3 mm, there was a 50% increase in σ*_int_* under the 100 N load, and then, there was a linear decrease to 0. Under the 1000 N load, the maximum stress in the deep wedge coincided with that in the shallow wedge. For the 0.1 and 0.2 mm rounded defects, the stresses in the deep wedge were lower. The maximum reductions occurred under the 100 N condition, and this was by half and under the 1000 N condition, and this was by 25%.

When using material 1 under a load level of 400 N, the deviations were smaller, indicating that the stress level in the dentin was lower when we were using this material in the case of the deep wedge.

The dependences of the maximum strain rates were similar to those that were found in the stress analysis. The differences in the deformation behaviour of the models with different wedge defect depths decreased as the load increased.

It may be assumed that in the deep wedges with a radius of 0.2 mm, the use of fillings was reasonable as it allowed for a significant reduction in the stress and strain levels in both the enamel and dentin. In this case, material 1 was preferred because the stress level in the enamel was reduced more rapidly.

## 4. Discussion

This paper presents the results of a preliminary study that was aimed at investigating the mechanical aspects of different ways of treating teeth with NCCLs. The numerical models of NCCLs is low in the scientific literature. They address various problems in the use of dental materials in restoring the surfaces of teeth. Studies of the means by which the thermal and mechanical properties influence of the restorative material on the choice of materials by dentists are carried out [39]. Ambiguous results are obtained when modelling the behaviour of the biomechanical units with materials different types. A small effect of the restorative material on the stresses in the tooth has been noted in previous works. This indicates the need to introduce complex models of the materials behaviour into practice [40]. The other authors’ results showed the effect of the restorative material and geometry on the restored tooth stress state, which were similar to those which were obtained by us [41].

Overall, the results that were obtained in this article are novel in terms of the quantitative analysis and comparison that was undertaken of the deformation behaviour of the teeth with and without a defect and having undergone a restoration. The study analysed the effect of the defect depth on the deformation behaviour of the biomechanical unit. The effect of the fillet radius and the type of restorative material on the overall stress state was evaluated. Overall, the results are consistent with clinical practice in the treatment of NCCLs, thereby substantiating popular clinical solutions in terms of solid mechanics. At this point, the geometric model of the tooth has the following limitations:

- The root system of the tooth in the model was replaced by kinematic boundary conditions;
- The influence of the gingiva was not taken into account;
- Enamel and dentin are deformed together;
- The contact interaction was modelled as a complete bonding of the mating surfaces.

The material models have the following limitations:

- The behaviour was described as being isotropically elastic;
- The polymerisation shrinkage of the materials was not considered.

Similar limitations are found in the models by other authors, e.g., [21].

The properties of the materials are taken for the dry state of them. To date, the mechanical properties of dental materials and cements have been extensively studied with regard to hydration [42,43]. Obviously, the consideration of the material properties with regard to their moisture content contributes to the deformation behaviour of the teeth and restorations. However, this issue is difficult to model, and it deserves a separate large-scale study.

The enamel–dentine bond was not considered in the model. According to the studies, its thickness is about 3 μm [44], which is very small for the construction of a 3D model of the crown of a tooth. In addition, the mechanical properties of this zone are difficult to describe within the currently accepted limitations of the model. The enamel–dentine bond in terms of its mechanics represents the natural bonding of the enamel and dentin to allow for joint deformation to occur [45,46]. In a numerical model, the joint deformation of two media with different mechanical properties is realised by a joint finite element mesh, according to the “node-to-node” principle. This principle is also implemented in our model.

The constraints that we have adopted can also be found in the models by other authors, e.g., [21].

Despite extensive practice in the treatment of wedge-shaped defects, there is currently no single solution. The multiple causes and development of NCCLs require comprehensive treatment. Some researchers report the need for orthodontic correction in conjunction with cavity closure [47]. The use of veneer onlays in conjunction with direct restorations is common [48]. However, it has been observed that up to 10% of restorations fall out or experience cracking during long-term follow-up studies [49]. The digital experiments use will allow the choosing of a material for restoration and treatment tactics based not only on clinical experience, but also on the numerical experimental results for a particular patient. The numerical experiment introduction into dental practice makes it possible to explore the applicability of the new materials for the restorations manufacture.

The growing development of multifunctional biomaterials with antimicrobial, remineralising and regenerative properties should be noted. Their development will provide new clinically effective dental materials to control the pathogens in the oral cavity and extend the life of dental restorations [50,51]. An important focus of research on new materials is to investigate their effect on the demineralisation of tissues at the tooth–restorative interface. The authors of [52] report that the use of the SPRG-filler-containing adhesive (i.e., FL Bond II) alone cannot effectively reduce the demineralisation of enamel, but if the SPRG-filler-containing composite is used in combination with it, then the cumulative synergy effect for anti-demineralisation becomes significantly higher. The restorations using a 10-MDP-containing adhesive (i.e., Single Bond Universal) with a composite resin filling appeared to reduce the demineralisation of the enamel, but it had no obvious effect when it was used on the root dentin. It is also worth noting that the composite resin containing the SPRG-filler (such as Beautifil II) performed exceptionally well in resisting acid attack, and thus, it has good potential for the prevention of recurrent caries. The preservation of tooth aesthetics for the psychological comfort of the patients also remains an important aspect in the study of new restorative materials. A discussion on the aesthetic effects of using the nanocomposite resin Z350 to restore anterior tooth defects was presented in [53].

The results of our study, which are presented in terms of the distribution and intensity of the stress in the tooth with the defect, are comparable with the results that were obtained by Jakupović et al. [8]. In the present study, the maximum stress on the boundary of the restoration and the tooth was observed near the acute end of the wedge or near the end of the lower surface of the restoration; a result which is consistent with the findings of [20]. An important finding of this article is the influence of the geometry of the defect and the restorative material on the stress state of the biomechanical system. Using the contact deformation mechanism of the tooth-restoration system approximates the nature of the deformation to real life cases. To date, studies on numerical analysis of the deformation of the tooth with restorations of the biomechanical system have shown that contact bonding has little effect, which does not allow for a reliable assessment of the deformation features of the biomechanical unit [19,23]. In the present paper, it was established that Herculite XRV is the most suitable material for deep wedging. It was found that increasing the fillet radius of the acute end of the “wedge” resulted in there being a more favourable stress–strain state of the biomechanical junction. However, the insertion of the restoration led to an increase in the level of maximum stresses in the tooth, but the local maxima were shifted to the zone of the kinematic boundary conditions. This effect requires us to further revise the boundary conditions and parameters of the tooth and the filling.

A new technique for the treatment of non-carious necks of teeth was proposed in [54]. The new restoration was designed with a V-shaped wedge defect. Creating an efficient inlay numerical simulation procedure for this type of wedge is much easier than it is for the U-shaped defects. The influence of U-shaped wedge-shaped defects of various configurations on the tooth stress–strain state is considered in this paper. The deformation evaluation of the U-shaped wedge-shaped defects with and without direct restorations is necessary due to their geometrically wide variety.

Thus, this study allowed us to collect the preliminary data on the influence of various factors on tooth deformation in U-shaped wedge-shaped defects before and after their restorations using standard methods.

## 5. Conclusions

In this study, in general, the installation of direct NCCL restorations on the main volume of dental materials demonstrated levels of deformation parameters that were comparable to a tooth without damage. The influence of the contact parameters and the kinematic conditions in eliminating the local zones of maximum stress and strain levels in the biomechanical unit needs to be investigated. The problem of the friction–contact interaction in the tooth-restorative contact region should be further studied with the specification of the finite-element model.

The Herculite XRV restorative material (Kerr Corp, Orange, CA, USA) was found to provide the biomechanical system with a favourable stress state in this formulation of the problem. It should be noted that deepening the defect towards the pulp was found to lead to higher stress and strain levels in the enamel and dentine. It is, therefore, important to treat NCCLs with suitable materials as early as possible and to ensure the longevity of the restorations by achieving a naturally occurring stressed state to prevent the development of the defect down the tooth.

## Figures and Tables

**Table 1:** Mechanical properties of tooth materials.

Parameter	Enamel	Dentin
E, MPa	72.7 × 10^3^	18.6 × 10^3^
v	0.33	0.31

**Table 2:** Mechanical properties of inlay materials.

Parameter	Material 1	Material 2
*E*, MPa	9.5 × 10^3^	14.1 × 10^3^
v	0.24	0.24

## Data Availability

Not applicable.
